# Isolated Diastolic Hypertension and Risk of Cardiovascular Events: A Systematic Review and Meta-Analysis of Cohort Studies With 489,814 Participants

**DOI:** 10.3389/fcvm.2021.810105

**Published:** 2022-01-05

**Authors:** Mingyan Huang, Linzi Long, Ling Tan, Aling Shen, Mi Deng, Yuxuan Peng, Wenwen Yang, Hongzheng Li, Yue Wei, Meng Li, Feifei Liao, Chao Liu, Aimei Lu, Hua Qu, Changgeng Fu, Keji Chen

**Affiliations:** ^1^Department of Cardiology, Xiyuan Hospital of China Academy of Chinese Medical Sciences, Beijing, China; ^2^National Clinical Research Center for Cardiovascular Diseases of Traditional Chinese Medicine, Beijing, China; ^3^Beijing University of Traditional Chinese Medicine Graduate School, Beijing University of Chinese Medicine, Beijing, China; ^4^Academy of Integrative Medicine, Fujian University of Traditional Chinese Medicine, Fuzhou, China; ^5^National Medical Products Administration (NMPA) Key Laboratory for Clinical Research and Evaluation of Traditional Chinese Medicine, Beijing, China

**Keywords:** isolated diastolic hypertension, cardiovascular events, cardiovascular mortality, stroke, meta-analysis, cohort study

## Abstract

**Background:** The association between isolated diastolic hypertension (IDH) and cardiovascular events has been inconsistently reported. This meta-analysis of cohort studies was designed to investigate the effect of the 2018 European Society of Cardiology (ESC) definition of IDH on the risk of composite cardiovascular events, cardiovascular mortality, all-cause mortality, and all strokes including ischemic stroke (IS) and hemorrhagic stroke (HS).

**Methods:** PubMed, Embase, the Cochrane Library, and Web of Science were searched from inception to July 6, 2021. Cohort studies that investigated the association between IDH and cardiovascular events risk, compared to normotension, were included. Pooled hazard ratios (HRs) and 95% CIs were calculated using a random-effects models and heterogeneity was evaluated using *Q*-test and *I*^2^ statistic. The robustness of the associations was identified using sensitivity analysis. The methodological quality of the studies was assessed using the Newcastle–Ottawa scale. Publication bias was assessed using funnel plot, trim-and-fill method, Begg's test, and Egger's test.

**Results:** A total of 15 cohort studies (13 articles) including 489,814 participants were included in this meta-analysis. The follow-up period ranged from 4.3 to 29 years. IDH was significantly associated with an increased risk of composite cardiovascular events (HR 1.28, 95% CI: 1.07–1.52, *p* = 0.006), cardiovascular mortality (HR 1.45, 95% CI: 1.07–1.95, *p* = 0.015), all strokes (HR 1.44, 95% CI: 1.04–2.01, *p* = 0.03), and HS (HR 1.64, 95% CI: 1.18–2.29, *p* = 0.164), but not associated with all-cause mortality (HR 1.20, 95% CI: 0.97–1.47, *p* = 0.087) and IS (HR 1.56, 95% CI: 0.87–2.81, *p* = 0.137). Subgroup analysis further indicated that IDH in the younger patients (mean age ≤ 55 years) and from Asia were significantly associated with an increased risk of composite cardiovascular events, while the elderly patients (mean age ≥ 55 years), Americans, and Europeans were not significantly associated with an increased risk of composite cardiovascular events.

**Conclusion:** This meta-analysis provides evidence that IDH defined using the 2018 ESC criterion is significantly associated with an increased risk of composite cardiovascular events, cardiovascular mortality, all strokes and HS, but not significantly associated with all-cause death and IS. These findings also emphasize the importance for patients with IDH to have their blood pressure within normal, especially in the young adults and Asians.

**Trial Registration:** PROSPERO, Identifier: CRD42021254108.

## Introduction

Isolated diastolic hypertension (IDH) is an important subtype of hypertension defined as a systolic blood pressure (SBP) of < 130 mm Hg and a diastolic blood pressure (DBP) of at least 80 mm Hg according to the 2017 American College of Cardiology (ACC)/American Heart Association (AHA) criterion (1) and an SBP of < 140 mm Hg with a DBP of at least 90 mm Hg according to the 2018 European Society of Cardiology (ESC) criterion (2). Compared with using the 2018 ESC guidelines, applying the 2017 ACC/AHA guidelines, it increased the prevalence of IDH from 1.3 to 6.5% in the United States (3), 7.79 to 24.72% in China (4), and 5.2 to 17.9% in Korea (5). However, IDH has usually been neglected and the treatment and awareness rates of this condition remain low. A previous study demonstrated that 86.1% of patients with IDH did not receive treatment and only 10.3% of untreated patients knew that they had hypertension (6). The number of deaths from cardiovascular events reached 17.7 million in 2017, accounting for approximately one-third of the total deaths (55 million) worldwide (7). Hypertension is the leading modifiable risk factor for cardiovascular events (8–10). The prognostic value of isolated systolic hypertension for cardiovascular events has been determined through a series of longitudinal clinical trials and meta-analysis studies (11–14), while there was only one meta-analysis reported that IDH diagnosed using the 2017 ACC/AHA criterion was not consistently associated with the cardiovascular disease (CVD) risk (15). However, whether IDH diagnosed using the 2018 ESC guidelines is associated with an increased risk of composite cardiovascular events remains controversial.

A recent prospective cohort study by Wu et al. indicated that IDH was associated with cerebral hemorrhage, myocardial infarction (MI), and total CVD compared to normotension (4). Conversely, McEvoy et al. demonstrated that IDH was not associated with the incidence of atherosclerotic CVD (3). Therefore, this systematic review and meta-analysis of published cohort studies were performed to further identify the association between IDH diagnosed using the 2018 ESC guideline and composite cardiovascular events, cardiovascular mortality, all-cause mortality, and all strokes including hemorrhagic stroke (HS) and ischemic stroke (IS).

## Methods

### Data Sources and Searches

The meta-analysis was performed according to the Preferred Reporting Items for Systematic Reviews and Meta-analyses (PRISMA) (16) and the Meta-analysis of Observational Studies in Epidemiology (MOOSE) guidelines (17), The protocol was registered in the International prospective register of systematic reviews (PROSPERO) (CRD42021254108). We conducted a meta-analysis of cohort studies that examined the association between IDH and the risk of cardiovascular events. Publications were identified by searching PubMed, Embase, the Cochrane Library, and Web of Science without language restrictions from inception to July 6, 2021. The following medical subject headings and free-text terms were searched (eAppendix 1): (hypertension or high blood pressure) and (diastole or isolated diastolic hypertension or IDH) and (cardiovascular diseases or cardiovascular events or cardiovascular deaths or cardiovascular or cardiac or myocardial ischemia or coronary artery disease or coronary heart disease or acute coronary syndrome or ischemic heart disease or myocardial infarction or heart failure or atrial fibrillation or stroke or cerebrovascular disorders or cerebrovascular accident or cerebrovascular disease or cerebrovascular or cerebral or complication or mortality or fatality or death) and (cohort studies or cohort or follow-up or observational or longitudinal or prospective). Additional articles were identified by manually searching the reference lists of pertinent articles.

### Study Selection

Studies were included if they met the following inclusion criteria: (1) cohort study; (2) performed in the general adult population (age > 18 years); (3) reported the associations of IDH with the composite of cardiovascular events, cardiovascular mortality, all-cause mortality, all-strokes, IS, and HS; (4) defined IDH and normotension based on the 2018 ESC guidelines (SBP < 140 mm Hg/DBP ≥ 90 mm Hg vs. SBP < 140 mm Hg/DBP < 90 mm Hg); and (5) reported hazard ratios (HRs) with corresponding 95% CIs for the association between IDH and cardiovascular events or sufficient data for their calculation. We excluded studies if they: (1) involved pregnant, critically ill, or hospitalized participants or (2) were published as comments, conference abstracts, or letters to the editor. When republished studies that included participants from the same cohort and reported similar outcome measures were found, articles reporting the most relevant data were selected. However, if duplicate studies provided information on different outcomes, they were included in the specific outcome analysis. Two investigators (MYH and HQ) independently screened all the titles or abstracts and reviewed the full texts to determine the eligibility of the identified studies and the validity of the extracted data. Any disagreements were resolved through a discussion or by a third reviewer (CGF).

### Data Extraction and Quality Assessment

Two investigators (MYH and LZL) independently extracted data from each eligible publication using a standardized data collection form. Any disagreements were resolved by consulting a third investigator (CGF). We used HRs to measure the associations. The primary outcomes of interest in this study were the composite of cardiovascular events. The secondary outcomes of interest were cardiovascular mortality, all-cause mortality, all strokes, IS, and HS. When an article was unavailable or to obtain additional information for analyses, an e-mail requesting the article or information was sent to the corresponding author. We recorded the following study characteristics: first author, publication year, study design, country, age at entry, percentage of male participants, cohort sample size, key exclusion criteria, outcomes, follow-up duration, treatment status at baseline, methods of BP measurement, ascertainment of outcomes, HRs and 95% CIs, and confounding variables adjusted in the multivariate analysis.

Two investigators (MYH and HQ) assessed the study quality using the Newcastle–Ottawa quality assessment scale for cohort studies (18). This scale allocated a total of nine points for the following three aspects: study selection (0–4 points), comparability (0–2 points), and ascertainment of the outcome of interest (0–3 points). We assigned scores of 0–3, 4–6, and 7–9 for low-, moderate-, and high-quality studies, respectively. Disagreements on quality assessment were resolved through a discussion with a third investigator (CGF).

### Statistical Analysis

Hazard ratios and 95% CIs were considered as the measure of the association between IDH and the cardiovascular event risk. All the studies included in the meta-analysis reported HRs and 95% CIs. We preferentially pooled the results from the multivariate-adjusted models with the most complete adjustment for underlying confounders. A random-effects model accounting for variation between studies was applied, as this can provide more conservative results than a fixed-effects model. We used Cochran's Q test (*p* < 0.10) to assess the heterogeneity among studies and *I*^2^ statistic to quantify the percentage of the total variation due to that heterogeneity. Low, moderate, and high heterogeneity were defined as *I*^2^ values of 0–25, 26–75, and > 75%, respectively (19). We then conducted random-effects subgroup analyses and sensitivity analyses to identify the sources of heterogeneity among studies and evaluate the robustness of the associations. Subgroup analyses were stratified by mean age, study location, treatment status at baseline, body mass index (BMI), and method of BP measurement. In sensitivity analyses, we used a leave-one-out method to observe the influence of individual studies on the overall risk estimate of HR. Potential publication bias was evaluated using visual assessment of funnel plots, Begg's test, and Egger's test. Potential adjustment for missing studies was approached by Duval and Tweedie trim-and-fill method. All the statistical analyses were performed using the Stata (version 15.0; Stata Corporation, College Station, Texas, USA). All the tests were two-sided and statistical significance was set at *p* < 0.05.

## Results

### Study Selection

**Figure 1:**
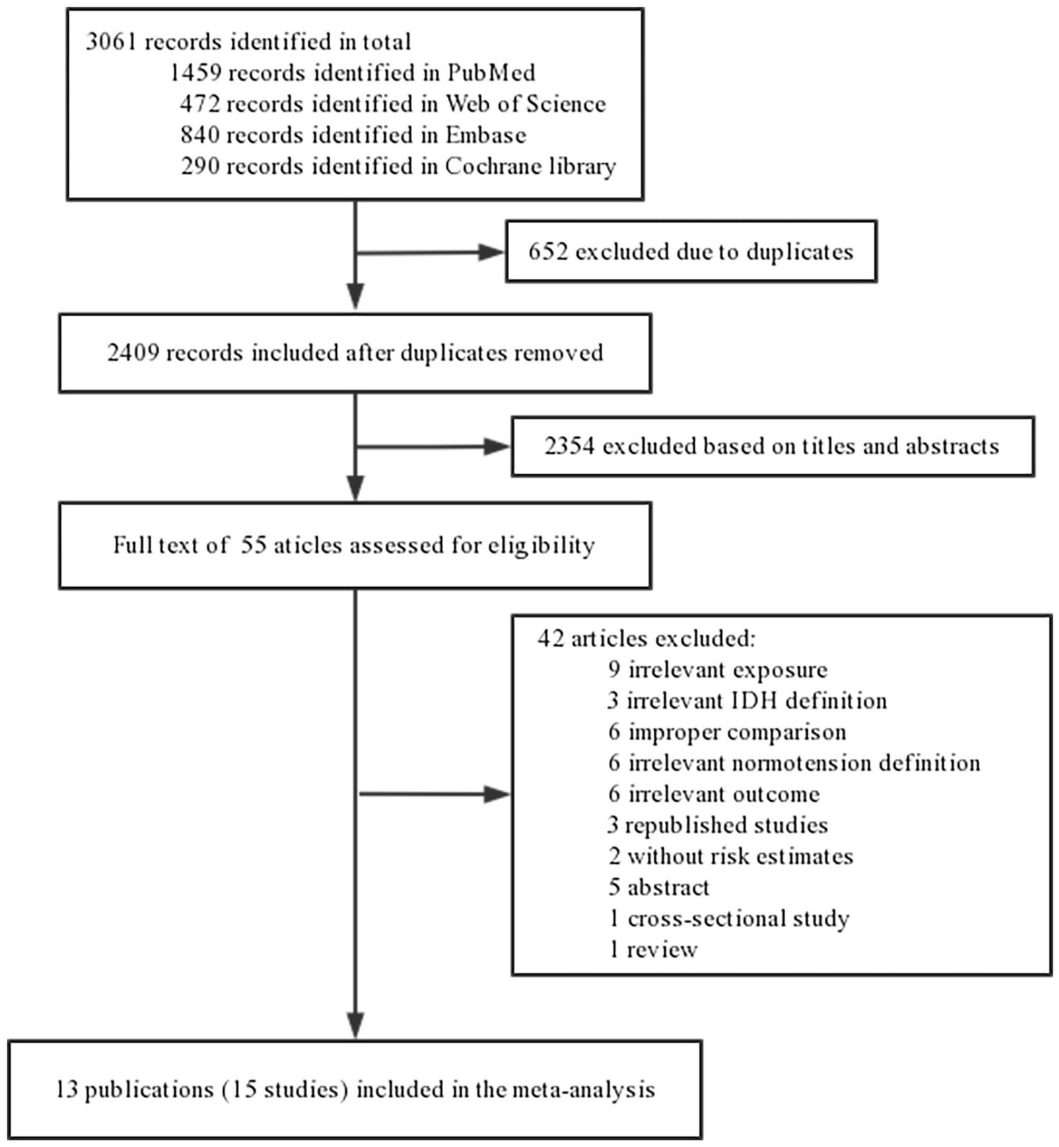
Flowchart of literature search and study selection.

Figure 1 shows the study selection process. We identified 1,459 articles from PubMed, 472 articles from Web of Science, 840 articles from Embase, and 290 articles from the Cochrane Library. After excluding 652 duplicates and 2,354 irrelevant articles based on titles and abstracts, 55 full articles remained for further examination. After a careful review of these records, 42 articles were excluded for the following reasons: IDH was not reported as the relevant exposure variable (*n* = 9), irrelevant IDH definition (*n* = 3), improper comparison (*n* = 6), irrelevant normotension definition (6), irrelevant study outcome (*n* = 6), republished studies (*n* = 3), studies not reporting HRs (*n* = 2), abstract-only articles (*n* = 5), cross-sectional studies (*n* = 1), and reviews (*n* = 1). Finally, 13 articles with 489,814 participants were included in the meta-analysis, as one publications included three independent cohort studies (3). Therefore, 15 studies from 13 articles were included in the meta-analysis.

### Characteristics of Included Studies

Table 1 shows the characteristics of the included cohort studies published between 2000 and 2021. Table 2 shows the characteristics of the participants and outcome ascertainment methods. Thirteen studies were prospective cohort studies and the other studies were retrospective cohort studies. The follow-up duration ranged from 4.3 to 29 years. Seven studies were conducted in Asia (four studies in China, two studies in Japan, and one study in Iran), four studies in the United States of America, and four studies in Europe (two studies in Finland, one study in the United Kingdom, and one study in Swedish). Overall, seven studies investigated the occurrence of composite cardiovascular events (coronary heart disease, strokes, and cardiovascular death in four studies; coronary heart disease and strokes in three studies; and coronary heart disease, strokes, heart failure, and cardiovascular death in one study), eight studies evaluated the risk of cardiovascular mortality, six studies assessed the risk of all-cause mortality, 4 studies evaluated the risk of all strokes, and 3 studies assessed the risk of IS and HS. All the studies performed adjustment for age. Most cohorts were controlled for some general risk factors including age (*n* = 15), smoking (*n* = 14), sex (*n* = 13), BMI (*n* = 11), diabetes mellitus (*n* = 10), alcohol consumption (*n* = 9), antihypertensive treatment (*n* = 7), hypercholesterolemia (*n* = 7), education (6), previous cardiovascular events (*n* = 5), SBP (*n* = 5), race (*n* = 4), physical exercise (*n* = 3), and other relative confounders. The results of the quality assessment based on the Newcastle–Ottawa Scale are shown in eTable 1. The overall quality of the included studies was high, with a median score of 8 (range, 7–9).

**Table 1:** Characteristics of studies included in the meta-analysis.

**References**	**Study design (cohort)**	**Country**	**No of participants (n)**	**Age (years), range, mean**	**Male sex (%)**	**Follow-up duration (years)**	**Study outcomes**
Hozawa et al. (20)	Prospective	Japan	1,492	≥40, 58.77	38.05	8.6	CV mortality
Fang et al. (21)	Prospective	China	18,787	≥35, 49.12	48.79	9.5	All strokes, IS, HS
Kelly et al. (22)	Prospective	China	128,752	≥40, 54.04	50.13	8.3	CV events, CV mortality
Barengo et al. (23)	Retrospective	Finland	13,537	25–64, 40.50	NA	16	CV mortality, all-cause mortality
Carlsson et al. (24)	Retrospective	Swedish	183	46–65	100	26	CV mortality
Carlsson et al. (24)	Retrospective	Swedish	173	46–65	0	26	CV mortality
Niiranen et al. (25)	Prospective	Finland	1,233	44–74, 53.8	41.6	11.2	CV events
Sun et al. (26)	Prospective	China	27,579	≥35, 48.25	50.73	4.3	All strokes, HS, IS
Lotfaliany et al. (27)	Prospective	Iran	5,959	30–64, 42.58	44.91	10.06	CV events, CV mortality, all-cause mortality
Lotfaliany et al. (27)	Prospective	Iran	425	≥65, 69.82	65.88	10.06	CV events, CV mortality, all-cause mortality
Hisamatsu et al. (28)	Prospective	Japan	1,474	30–49, 38.15	66	29	CV mortality
McEvoy et al. (3)	Prospective	USA	10,540	46–69, 54.54	43.24	25.2	CV events
McEvoy et al. (3)	Prospective	USA	43,097	≥20, 40	NA	9.8	CV mortality, all-cause mortality
McEvoy et al. (3)	Prospective	USA	17,687	≥20, 42	NA	28.7	CV mortality, all-cause mortality
McGrath et al. (29)	Prospective	UK	151,831	37–70, 54	40	9.8	CV events, all strokes
Jacobsen et al. (15)	Prospective	USA	5,104	45–84, 60.46	49	13	CV events, all-cause mortality
Wu et al. (4)	Prospective	China	61,961	18–98, 48.72	77	10.41	CV events, all-cause mortality, IS, HS

*CV, cardiovascular; HS, hemorrhagic stroke; IS, ischemic stroke; UK, United Kingdom; USA, United States of America*.

**Table 2:** Characteristics of included participants and outcome ascertainment.

**References**	**Treatment status at baseline**	**BP measurement method**	**Key exclusion criteria**	**Outcome ascertainment**	**Adjusted covariates**
Hozawa et al. (20)	Combined	The average of 2 readings taken by a nurse or technician with the subjects seated, after at least 2 min of rest, using a semiautomatic device	Dementia or bedridden status and out-of-town work	Defined as death from disease of the circulatory system based on ICD-10	Age, sex, smoking, obesity, antihypertensive treatment, previous CVD, hypercholesterolemia, and diabetes mellitus
Fang et al. (21)	Combined	Seated BP measured twice in the right arm with standard sphygmomanometer by clinic personnel	Previous stroke	Diagnosed by neurologists following the World Health Organization Monitoring Trends and Determinants in Cardiovascular Disease criteria	Age, BMI, smoking, drinking, and history of heart disease
Kelly et al. (22)	Untreated	The average of 2 readings taken by a trained observer with the subjects seated quietly for 5 min, using a standard mercury sphygmomanometer	Missing BP values and prevalent CVD, CHD, or stroke	Events investigated and validated using hospital records, death certificates, and interviews, and classified according to ICD-9	Age, sex, education, smoking, drinking, physical inactivity, BMI, geographic region, urbanization, and diabetes
Barengo et al. (23)	Treated	BP measured twice from the right arm of the participant in sitting position after at least a 5-min rest using a standard mercury sphygmomanometer	Previous CHD, HF, or cancer, or incomplete data at baseline	Record linkage to the nationwide death register of the Statistics of Finland coded according to ICD-10	Age, sex, region, study year, education, history of diabetes, smoking, cholesterol, BMI, and physical activity
Carlsson et al. (24)	NA	BP measured manually with the participant in a supine position after 20 min of rest	NA	Record linkage to the Swedish National Cause-of-Death Register and physician-issued certificates coded according to ICD-8/9	Age
Niiranen et al. (25)	Combined	The average of 2 office BP measured by a nurse using mercury sphygmomanometer from the sitting individual's right arm after a 10-min rest.	Previous CVD	Ascertained through linkage to the National Hospital Discharge Register and the nationwide Causes-of-Death Register coded according to ICD-10	Age, sex, smoking, antihypertensive treatment, previous CVD, hypercholesterolemia, and diabetes mellitus
Sun et al. (26)	Combined	Seated BP measured three times by a trained and certified observer using a standardized electronic sphygmomanometer after a 5-min rest.	Suffering stroke at baseline, a history of tumors, HF or pregnancy	Ascertained through home visits, hospital records, autopsy reports, death certificates coded according to ICD-9	Antihypertensive treatment, age, sex, BMI, smoking, drinking, diabetes, lipid disorder, CHD, and SBP
Lotfaliany et al. (27)	Untreated	Two measurements of BP were performed using a standardized mercury sphygmomanometer on the right arm after a 15-min rest in a sitting position.	Previous CVD	Followed up annually for any medical event and death certificate	Age, sex, smoking, diabetes status, hypercholesterolemia, low HDL, and BMI
Hisamatsu et al. (28)	Untreated	BP was measured by trained public health nurses using a standard mercury sphygmomanometer on the right arm of seated participants after at least 5 min of rest.	CVD, use of antihypertensive medications	Linkage to the National Vital Statistics database of Japan coded according to ICD-9/10	Age, sex, smoking, drinking, BMI, total cholesterol, and diabetes mellitus
McEvoy et al. (3)	Combined	BP was measured after 5 min of rest in the sitting position. We recorded BP as the mean of the last 2 of 3 measurements collected over 5-min intervals	Previous CVD	Confirmed using hospital discharge records and death certificates	Age, sex, race, education, smoking, drinking, HDL, LDL, triglycerides, eGFR, BMI, antihypertensive treatment, diabetes, SBP and pulse pressure
McEvoy et al. (3)	NA	NA	Missing data on relevant variables of interest or SBP	Follow-up of participants continued until any death or cardiovascular death coded according to ICD-10	Age, sex, race, smoking, drinking, BMI, total cholesterol, lipid-lowering medications, diabetes, and SBP
McEvoy et al. (3)	NA	BP was taken 3 times in the sitting position and the third reading was used for analyses.	Missing blood pressure measurements	Confirmed using the National Death Index, Maryland death certificates, local newspaper obituaries, and reports by next of kin	Age, sex, race, education, smoking, antihypertensive treatment, and SBP
McGrath et al. (29)	Combined	The average of two BP measurements were taken after 5 min in the seated position using an automatic digital BP monitor.	Systolic hypertension or baseline CVD	Linkage to national hospital records, death registrations, and primary care diagnoses	Age, sex, education, socioeconomic status, enrolment center, smoking, drinking, HDL, LDL, triglycerides, diabetes mellitus, BMI, antihypertensive treatment, eGFR, and SBP
Jacobsen et al. (15)	Combined	BP was measured three times after 5 min of seated rest using an automated oscillometric sphygmomanometer, and the mean of the last two measurements was used for analyses.	Systolic hypertension at baseline	Regular interview and self-reported diagnoses were verified using medical records and death certificates, and independently classified by two physicians	Age, sex, race, BMI, smoking, drinking, LDL, HDL, triglycerides, eGFR, antihypertensive medications, history of diabetes, and SBP
Wu et al. (4)	Untreated	The average of three BP measurements were taken, we obtained three readings at 5-min intervals after the participants had rested for at least 5 min.	Use of antihypertensive medications at baseline	Adjudicated through medical records from the Municipal, Social Insurance Institution and the Hospital Discharge Register by three experienced masked physicians	Age, sex, smoking, alcohol drinking, physical activity, education, income level, previous CVD, BMI, fasting serum glucose, TC, serum uric acid, CRP, and eGFR, and SBP

*BMI, body mass index; BP, blood pressure; CHD, coronary heart disease; CRP, C-reactive protein; CVD, cardiovascular disease; eGFR, estimated glomerular filtration rate; HDL, high-density lipoprotein; HF, heart failure; IDH, isolated diastolic hypertension; ICD, International Classification of Diseases; LDL, low-density lipoprotein; NHIS, National Health Insurance Service; SBP, systolic blood pressure; TC, total cholesterol*.

### Primary Outcomes

#### Composite Cardiovascular Events

**Figure 2:**
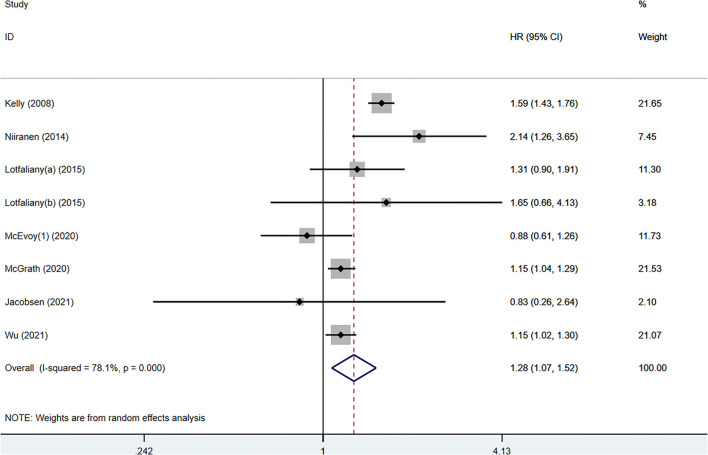
Forest plot of hazard ratios (HRs) for the association between isolated diastolic hypertension and cardiovascular event risk.

Seven studies with 365,805 participants showed a significant association between IDH and composite cardiovascular events (HR 1.28, 95% CI: 1.07–1.52, *p* = 0.006) compared to normotension (Figure 2) with significant heterogeneity (*I*^2^ = 78.1%, *p* < 0.001). In sensitivity analyses, the summary HRs ranged from 1.17 (95% CI: 1.04–1.32) to 1.34 (95% CI: 1.12–1.60) when individual studies were excluded from the analysis (eTable 2). Therefore, no individual study had a significant impact on the overall results. Funnel plots did not exhibit a notable publication bias and no evidence of publication bias based on Egger's test (*p* = 0.903) or Begg's test (*p* = 0.536) was found (eFigures 1A–C).

### Secondary Outcomes

#### Cardiovascular Mortality

The relationship between IDH and the cardiovascular mortality risk was evaluated in 8 studies with 212,779 participants. The pooled HR showed a significant association between IDH and cardiovascular mortality (HR 1.45, 95% CI: 1.07–1.95, *p* = 0.015), with moderate heterogeneity (*I*^2^ = 71.4%, *p* < 0.001) across the studies (eFigure 2). No evidence of publication bias was found using Egger's test (*p* = 0.504) or Begg's test (*p* = 0.210) and no asymmetry was observed in the funnel plots (eFigure 3).

#### All-Cause Mortality

Six studies with 147,770 participants were included in the meta-analysis of IDH and the risk of all-cause mortality (eFigure 4). However, the result showed that the association between IDH and the risk of all-cause mortality was not significant (HR 1.20; 95% CI: 0.97–1.47, *p* = 0.087), with moderate heterogeneity (*I*^2^ = 73.4%, *p* = 0.001). Visual inspection of the funnel plot indicated mild asymmetry. This was further confirmed by a significant Egger's test (*p* = 0.048), while the *p*-values of Begg's test were statistically non-significant (*p* = 0.230). Two missing studies were imputed in the contour-enhanced funnel plots and the application of the trim-and-fill method did not change the risk estimate (HR 1.07; 95% CI: 0.86–1.33, *p* = 0.566) (eFigures 5A–D).

#### Strokes

Four studies with 260,158 participants evaluated the association between IDH and all strokes (eFigure 6), demonstrating a significant association (HR 1.44, 95% CI: 1.04–2.01, *p* = 0.03). Three studies with 108,327 participants assessed the association between IDH and IS (eFigure 7), but not demonstrated a significant association (HR 1.56, 95% CI: 0.87–2.81, *p* = 0.137). Three studies with 108,327 participants evaluated the association between IDH and HS (eFigure 8), indicating a significant association (HR 1.64, 95% CI: 1.18–2.29, *p* = 0.003).

### Subgroup Analyses

Subgroup analyses stratified by mean age at entry, location, treatment status at baseline, BMI, and method of BP measurement were performed. IDH was significantly associated with an increased risk of composite cardiovascular events in most subgroups (Table 3), except for the average age of participants ≥ 55 years (HR 1.27, 95% CI: 0.62–2.60, *I*^2^ = 0, *P*_heterogeneity_ = 0.362), participants from America (HR 0.88, 95% CI: 0.62–1.24, *I*^2^ = 0, *P*_heterogeneity_ = 0.925) and Europe (HR 1.48, 95% CI: 0.81–2.70, *I*^2^ = 80.1%, *P*_heterogeneity_ = 0.025). Meta-regression analysis showed significant correlations between methods of BP measurement (*p* < 0.001). The source of heterogeneity among studies on the composite cardiovascular events may be due to the methods of BP measurement. The heterogeneity disappeared in groups when BP was measured in mercury sphygmomanometers (*I*^2^ < 0.1%, *P*_heterogeneity_ = 0.528) and automatic digital BP monitor (*I*^2^ < 0.1% *P*_heterogeneity_ = 0.583). Therefore, the source of heterogeneity among studies on the composite cardiovascular events may be due to the methods of BP measurement.

**Table 3:** Subgroup analyses of hazard ratios for the association between isolated diastolic hypertension (IDH) and composite cardiovascular events.

**Variable**	**No of studies**	**HR (95% CI)**	***I*** **^2^ (%)**	***P*** **-value for heterogeneity**
Mean age (years)^*^				
<55	6	1.28 (1.06, 1.54)	83.9	<0.001
≥55	2	1.27 (0.62, 2.60)	0	0.362
**Location**				
Asia	3	1.36 (1.07, 1.73)	81.3	0.001
America	2	0.88 (0.62, 1.24)	0	0.925
Europe	2	1.48 (0.81, 2.70)	80.1	0.025
**Treatment status at baseline**				
untreated	3	1.36 (1.07, 1.73)	81.3	0.001
combined	4	1.18 (0.86, 1.62)	60.7	0.054
**BMI (kg/m^2^)**				
<28	3	1.34 (1.04, 1.74)	87.4	<0.001
≥28	4	1.13 (1.02, 1.25)	0	0.415
**Method of BP measurement**				
Mercury sphygmomanometers	3	1.59 (1.44, 1.75)	0	0.528
Automatic digital BP monitor	2	1.15 (1.03, 1.28)	0	0.583
NA	2	1.06 (0.84, 1.35)	46.8	0.170

*BMI, body mass index; BP, blood pressure; HR, hazard ratio*.

* *Study by Lotfaliany et al., reported HRs stratified by middle-aged and the elderly persons*.

## Discussion

To the best of our knowledge, this is the first quantitative meta-analysis investigating the associations between the 2018 ESC definition of IDH and composite cardiovascular events, cardiovascular mortality, all-cause mortality, all strokes, IS, and HS. In this meta-analysis with 15 cohort studies involving 489,814 participants, three main findings emerged. First, the pooled results indicated that IDH is associated with an increased risk of composite cardiovascular events, cardiovascular mortality, all strokes, and HS, but not for all-cause mortality and IS. Second, IDH in the younger patients (mean age ≤ 55 years) was associated with an increased risk of composite cardiovascular events, but not in the elderly patients (mean age ≥ 55 years). Third, patients with IDH in Asia were significantly associated with an increased risk of composite cardiovascular events, while Americans and Europeans were not significantly associated with an increased risk of composite cardiovascular events.

The previous meta-analysis showed that IDH defined according to the 2017 ACC/AHA criterion was not consistently associated with new-onset CVD and the relative size of any potential association was slight (15). While in our meta-analysis, we found that the 2018 ESC definition of IDH was significantly associated with an increased risk of composite cardiovascular events. The discrepancy may be due to the difference in the definitions of IDH and the age of population. As also pointed by Jacobsen et al., people with IDH were at higher risk of composite cardiovascular events only when the DBP ≥ 90 mm Hg (15). The Hypertension Optimal Treatment (HOT) trial supported the point that the DBP value between 80 and 90 mm Hg had no adverse prognostic clinical importance, if the SBP was within the normal range and reported that a strategy of reducing the DBP to 80 mmHg was irrelevant to significant help in reducing the end point events, compared with lowering DBP to 90 mm Hg (30). In addition, the previous meta-analysis mainly focused on middle-aged or elderly people and may not apply to adults younger than 40 years, while our meta-analysis population included the age span of 18–98 years. Clinical and observational studies have proved elevated SBP as a more powerful predictor of the adverse cardiovascular outcomes than DBP in the elderly patients (11). However, younger subjects should not be ignored, as DBP instead of SBP was associated with composite cardiovascular events (31).

Furthermore, we performed subgroup analyses stratified by age, BMI, geographic location, treatment status at baseline, and methods of BP measurement. Some significant findings were obtained. The relationship between IDH and the risk of cardiovascular events varies according to age, geographic location, and treatment status at baseline. In our subgroup analyses by location, a significant association between IDH and an increased risk of composite cardiovascular events was found in Asia, mainly in China. However, no statistically significant association was found between American and European populations. This was an interesting finding and the observed discrepancy in this study was partly related to lower awareness and treatment rate among patients with IDH in China. On account of the traditional concept that “SBP matters most” in association with cardiovascular events and hypertension mostly asymptomatic (32), the awareness and treatment rate of IDH is low. Data from the National Health and Nutrition Examination Survey (NHANES) III cohort in America demonstrated that awareness among patients with IDH (46.8%) was significantly lower than patients with isolated systolic hypertension (ISH) (58.4%) and combined systolic and diastolic hypertension (SDH) (67.2%) (33). Data from the China PEACE Million Persons Project indicated that awareness among patients with IDH was 10.3% and that 86.1% of these were untreated (6). Moreover, the observed discrepancy may also reflect the genetic susceptibility and lifestyle differences between different regions (34–37). Thus, these results provide some clues for future studies on the biological mechanism between IDH and composite cardiovascular events among different ethnic backgrounds.

Isolated diastolic hypertension results from an increase in peripheral vascular resistance and is more prevalent in young and middle-aged adults (6, 22, 38–40). Chrysant observed that IDH was associated with an adverse cardiovascular events in younger patients (32). Similarly, a significant association between IDH and an increased risk of composite cardiovascular events was found in younger people (mean age < 55 years), but not in the elderly people (mean age ≥ 55 years). Fang et al. demonstrated that the prevalence of IDH was 8% in the 35–59 years age group and 4% in the elderly group (21). Similarly, Berney et al. confirmed that cardiovascular events are significantly related to SBP and pulse pressure in the elderly people, but are mainly related to DBP in younger people (41). Furthermore, our findings were consistent with those of a previous study that indicated that the impact of IDH on cardiovascular events and mortality was stronger in younger adults (age < 60 years) (42). Therefore, more attention should be paid to younger patients with IDH.

Furthermore, whether IDH needs treatment is controversial and the treatment rate of IDH is low (6). In our subgroup analysis by baseline treatment status, studies including only untreated participants at baseline showed that IDH was associated with an increased risk of composite cardiovascular events, whereas studies incorporating participants with treatment at baseline did not show this association. The results of our meta-analysis indicate that active treatment of IDH is helpful in reducing the risk of long-term composite cardiovascular events. However, there is no evidence from clinical trials on the efficacy of antihypertensive medications on BP reduction and long-term cardiovascular events in IDH. Therefore, clinical trials of antihypertensive medications are warranted to determine the effects on IDH.

This systematic review and meta-analysis had a number of strengths. First, the meta-analysis included close to 500,000 participants, providing sufficient statistical power to detect associations between IDH and cardiovascular events. Second, this meta-analysis was based on several cohort studies from various populations such as Asian, American, and European, which strengthened the generalizability of the findings. Third, the inclusion of cohort studies ensured that the exposure preceded the outcome, reduced the potential selection bias, and avoided recall bias. Furthermore, all of the included studies were of high quality and sensitivity analyses, further ascertained the robustness of the results.

## Limitations

This meta-analysis had several limitations. First, the definition of composite cardiovascular events was somewhat inconsistent in the included studies, which led to some bias; however, we defined the composite cardiovascular events as coronary heart disease, strokes, heart failure, and/or cardiovascular mortality; all data in this study were extracted according to the definition. Second, there was significant between-study heterogeneity as well. When performing subgroup analysis based on the measurement methods of BP for the composite cardiovascular events, the heterogeneity in the mercury sphygmomanometers group and the automatic digital BP monitor group disappeared and the results remained consistent. In consequence, the measurement methods of BP may be the main source of heterogeneity. Mercury sphygmomanometers and automatic digital BP monitors are two main methods of measuring DBP, while the mechanisms differ slightly for DBP (mercury sphygmomanometers being based on human auscultation and automatic digital BP monitors being based on algorithms that detect vibrations in the arterial wall). Previous study indicated that the automatic BP device may underestimate DBP by up to 3 mm Hg (43). Finally, although we extracted the maximum fully adjusted risk estimate, the adjusted confounders are not exactly the same in the included studies. Differential adjustment for confounders across different studies could potentially influence this study.

## Conclusion

This meta-analysis provides evidence that IDH defined using the 2018 ESC criterion is significantly associated with an increased risk of composite cardiovascular events, cardiovascular mortality, all strokes, and HS, but not significantly associated with all-cause death and IS. Subgroup analysis further indicates that the correlation between IDH and composite cardiovascular events is significant in younger people and Asians. Therefore, further studies are needed to clarify the age-stratified associations of IDH with cardiovascular events and attach importance to the young population and Asians with IDH. Furthermore, the results of subgroup analysis stratified by treatment status at baseline indicate that active treatment of IDH is helpful in reducing the risk of long-term composite cardiovascular events. However, there is no evidence from clinical trials on the efficacy of antihypertensive medications on BP reduction and long-term cardiovascular events in IDH. In consequence, future studies are needed to assess the impacts and cost-effectiveness of non-pharmacological and pharmacological treatments of IDH for reducing the risk of cardiovascular events.

## Data Availability Statement

The original contributions presented in the study are included in the article/Supplementary Material, further inquiries can be directed to the corresponding author/s.

## Author Contributions

All authors listed have made a substantial, direct, and intellectual contribution to the work and approved it for publication.

## Funding

This study was supported by the Youth Talent Promotion Project of China Association for Science and Technology (No.2020-QNRCI-02) and the Science and Technology Innovation Project of China Academy of Chinese Medical Sciences (NO.CI2021A05013).

## Conflict of Interest

The authors declare that the research was conducted in the absence of any commercial or financial relationships that could be construed as a potential conflict of interest.

## Publisher's Note

All claims expressed in this article are solely those of the authors and do not necessarily represent those of their affiliated organizations, or those of the publisher, the editors and the reviewers. Any product that may be evaluated in this article, or claim that may be made by its manufacturer, is not guaranteed or endorsed by the publisher.
