# Supplementary material for: Isolated Diastolic Hypertension and Risk of Cardiovascular Events: A Systematic Review and Meta-Analysis of Cohort Studies With 489,814 Participants
Source: Front Cardiovasc Med. 2022 Jan 5;8:810105. doi: 10.3389/fcvm.2021.810105 (PMC8766994; doi:10.3389/fcvm.2021.810105)
Supplement: Supplementary file 1 [file Data_Sheet_1.docx]

**eAppendix 1: literature search strategy for the databases**

**1. Search strategy for PubMed from inception to** **6^th^, July, 2021**

((((((cohort studies[MeSH Terms]) OR (Cohort[Title/Abstract])) OR (follow up[Title/Abstract])) OR (observational[Title/Abstract])) OR (longitudinal[Title/Abstract])) OR (prospective[Title/Abstract])) AND ((("Hypertension"[MeSH Terms] OR "Hypertension"[Title/Abstract] OR "high blood pressure"[Title/Abstract]) AND ("Diastole"[MeSH Terms] OR "Diastole"[Title/Abstract] OR "isolated diastolic hypertension"[Title/Abstract] OR "IDH"[Title/Abstract]) AND ("cardiovascular diseases"[MeSH Terms] OR "heart failure"[MeSH Terms] OR "Stroke"[MeSH Terms] OR "cerebrovascular disorders"[MeSH Terms] OR "cardiovascular disease"[Title/Abstract] OR "cardiovascular events"[Title/Abstract] OR "cardiovascular deaths"[Title/Abstract] OR "Cardiovascular"[Title/Abstract] OR "Cardiac"[Title/Abstract] OR "heart failure"[Title/Abstract] OR "myocardial ischemia"[Title/Abstract] OR "coronary artery disease"[Title/Abstract] OR "coronary heart disease"[Title/Abstract] OR "acute coronary syndrome"[Title/Abstract] OR "ischaemic heart disease"[Title/Abstract] OR "atrial fibrillation"[Title/Abstract] OR "Stroke"[Title/Abstract] OR "cerebrovascular accident"[Title/Abstract] OR "cerebrovascular disease"[Title/Abstract] OR "cerebrovascular disorder"[Title/Abstract] OR "Cerebrovascular"[Title/Abstract] OR "Cerebral"[Title/Abstract] OR "Complication"[Title/Abstract] OR "Mortality"[Title/Abstract] OR "Fatality"[Title/Abstract] OR "Death"[Title/Abstract] OR "myocardial infarction"[Title/Abstract])))

**2. Search strategy for Embase from inception to 6^th^, July, 2021**

#1 'hypertension'/exp OR hypertension:ab,ti OR 'high blood pressure':ab,ti

#2 'diastole'/exp OR diastole:ab,ti OR 'isolated diastolic hypertension':ab,ti OR idh:ab,ti

#3 'cardiovascular diseases'/exp OR 'cardiovascular disease':ab,ti OR 'cardiovascular events':ab,ti OR 'cardiovascular deaths':ab,ti OR cardiovascular:ab,ti OR cardiac:ab,ti OR 'heart failure':ab,ti OR 'myocardial ischemia':ab,ti OR 'myocardial infarction':ab,ti OR 'coronary artery disease':ab,ti OR 'coronary heart disease':ab,ti OR 'acute coronary syndrome':ab,ti OR 'ischaemic heart disease':ab,ti OR 'atrial fibrillation':ab,ti OR stroke:ab,ti OR 'cerebrovascular disorder':ab,ti OR 'cerebrovascular accident':ab,ti OR 'cerebrovascular disease':ab,ti OR cerebral:ab,ti OR cerebrovascular:ab,ti OR complication:ab,ti OR fatality:ab,ti OR mortality:ab,ti OR death:ab,ti OR 'heart failure'/exp OR 'stroke'/exp OR 'cerebrovascular disorders'/exp

#4 'cohort studies'/exp OR cohort:ab,ti OR 'follow up':ab,ti OR observational:ab,ti OR longitudinal:ab,ti OR prospective:ab,ti

**3. Search strategy for Web of science from inception to 6^th^, July, 2021**

#1 TS=(Hypertension OR high blood pressure)

#2 TS=(diastole OR isolated diastolic hypertension OR IDH)

#3 TS=(Cardiovascular Disease OR Cardiovascular events OR Cardiovascular deaths OR Cardiovascular OR Cardiac OR heart failure OR Myocardial Ischemia OR myocardial infarction OR coronary artery disease OR coronary heart disease OR acute coronary syndrome OR ischaemic heart disease OR atrial fibrillation OR Stroke OR cerebrovascular disorder OR cerebrovascular accident OR cerebrovascular disease OR Cerebral OR Cerebrovascular OR complication OR Fatality OR Mortality OR Death)

#4 TS=(cohort studies OR Cohort OR follow up OR observational OR longitudinal OR prospective)

#5 ： #1 AND #2 AND #3 AND #4

**4. Search strategy for Cochrane Library from inception to 6^th^, July, 2021**

#1 MeSH descriptor: [Hypertension] explode all trees

#2 (hypertension):ti,ab OR (high blood pressure):ti,ab

#3 #1 OR #2

#4 MeSH descriptor: [Diastole] explode all trees

#5 (isolated diastolic hypertension):ti,ab OR (IDH):ti,ab OR ( diastole):ti,ab

#6 #4 OR #5

#7 MeSH descriptor: [Cardiovascular Diseases] explode all trees

#8 MeSH descriptor: [Heart Failure] explode all trees

#9 MeSH descriptor: [Stroke] explode all trees

#10 MeSH descriptor: [Cerebrovascular Disorders] explode all trees

#11 (Cardiovascular Disease):ti,ab OR (Cardiovascular events):ti,ab OR (Cardiovascular deaths):ti,ab OR (Cardiovascular):ti,ab OR (Cardiac):ti,ab OR (heart failure):ti,ab OR (Myocardial Ischemia):ti,ab OR (myocardial infarction ):ti,ab OR (coronary artery disease):ti,ab OR (coronary heart disease):ti,ab OR (acute coronary syndrome):ti,ab OR (ischaemic heart disease):ti,ab OR (atrial fibrillation):ti,ab OR (Stroke):ti,ab OR (cerebrovascular disorder):ti,ab OR (cerebrovascular accident):ti,ab OR (cerebrovascular disease):ti,ab OR (Cerebral):ti,ab OR (Cerebrovascular):ti,ab OR (complication):ti,ab OR (Fatality):ti,ab OR (Mortality):ti,ab OR (Death):ti,ab 303271

#12 #7 OR #8 OR #9 OR #10 OR #11

#13 MeSH descriptor: [Cohort Studies] explode all trees

#14 (cohort):ti,ab OR (follow up):ti,ab OR (observational):ti,ab OR (longitudinal):ti,ab OR (prospective):ti,ab

#15 #13 OR #14

#16 #3 AND #6 AND #12 AND #15

**eFigure 1A-C.**

Egger’s test (A), Begg’s test (B) and funnel plot (C) for studies of isolated diastolic hypertension in relation to the risk of composite cardiovascular events.

A


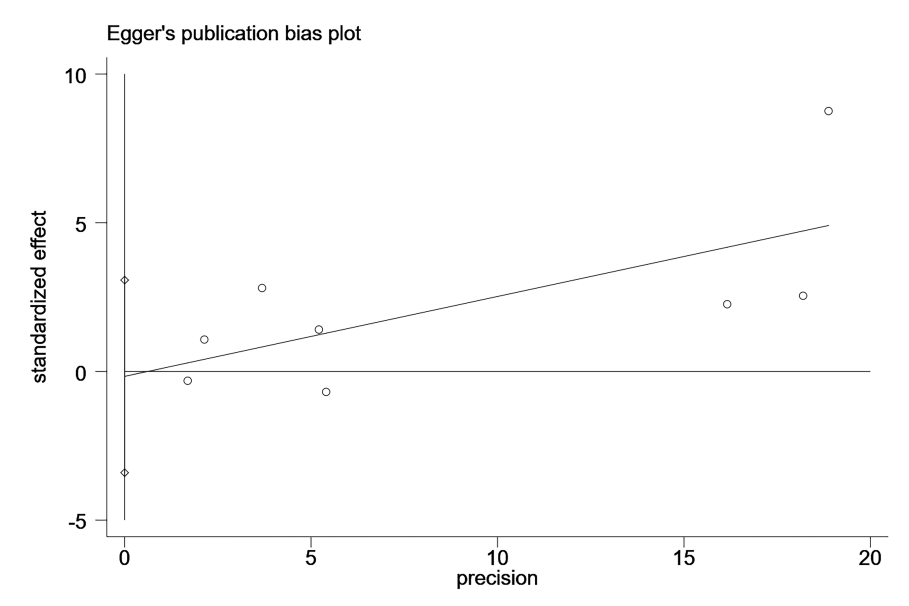


B


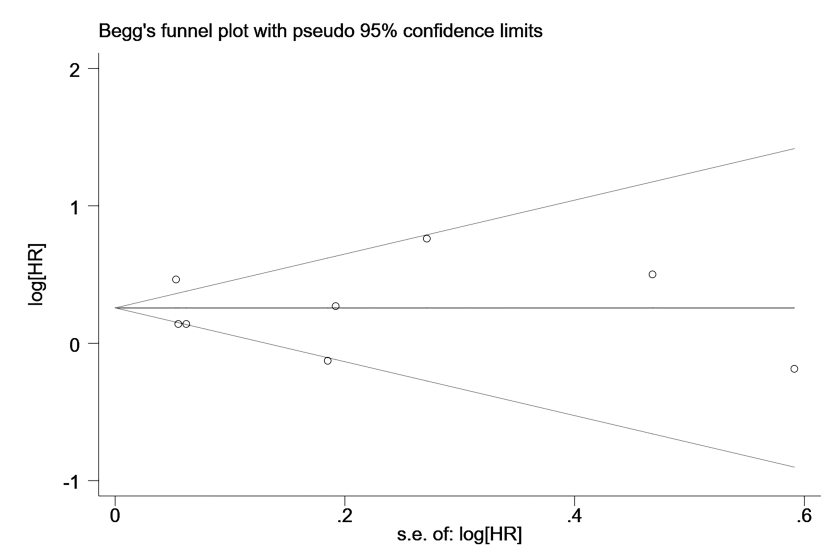


C


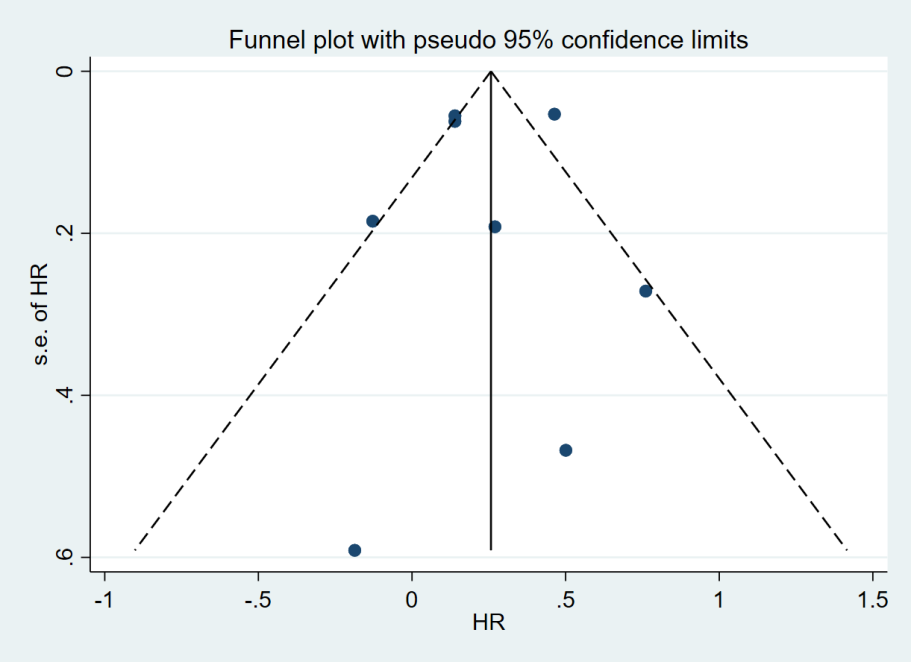


Abbreviations: HR, hazard ratio.

**eFigure 2. Forest plot of hazard ratios for the association between isolated diastolic hypertension and cardiovascular mortality risk**

**
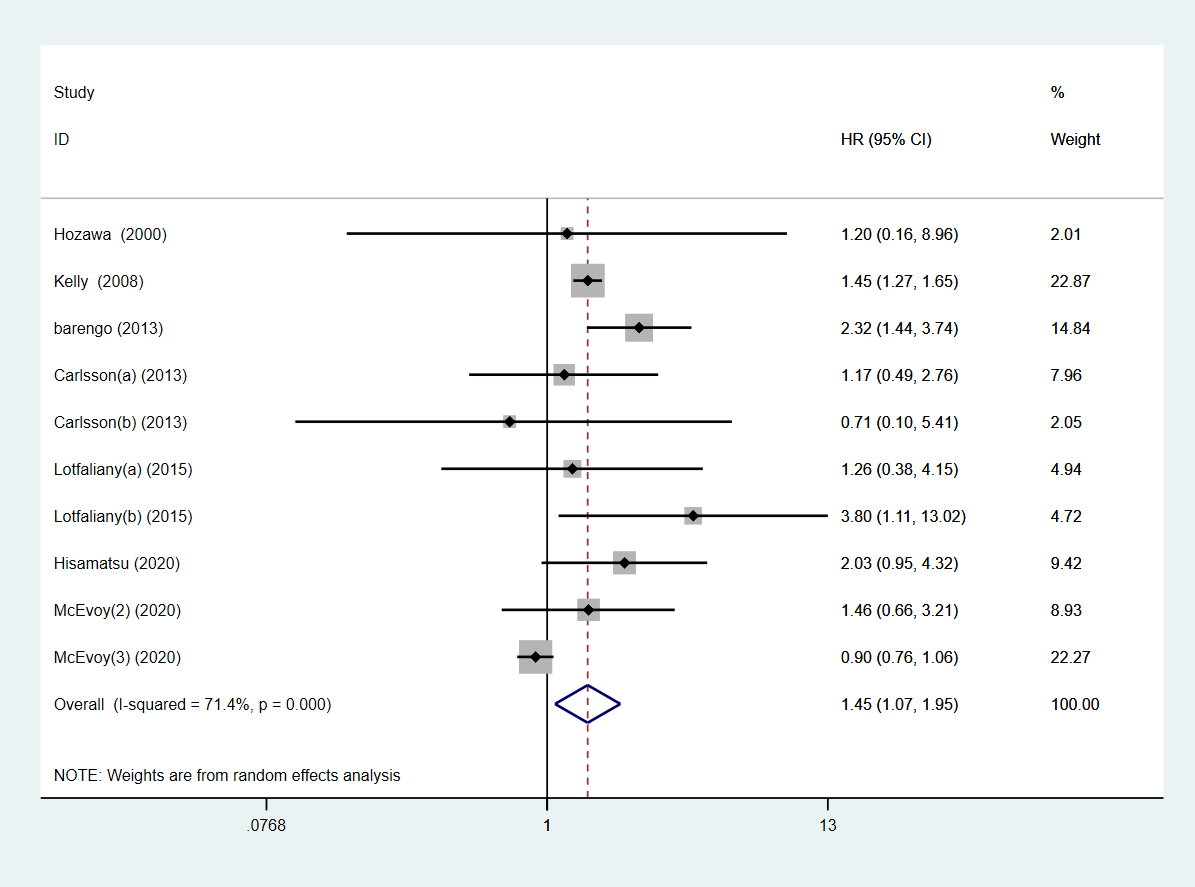
**

Abbreviations: CI, confidence interval; HR, hazard ratio.

**eFigure 3 Egger’s test (A), Begg’s test (B) and funnel plot (C) for studies of isolated diastolic hypertension in relation to the risk of cardiovascular mortality.**

A


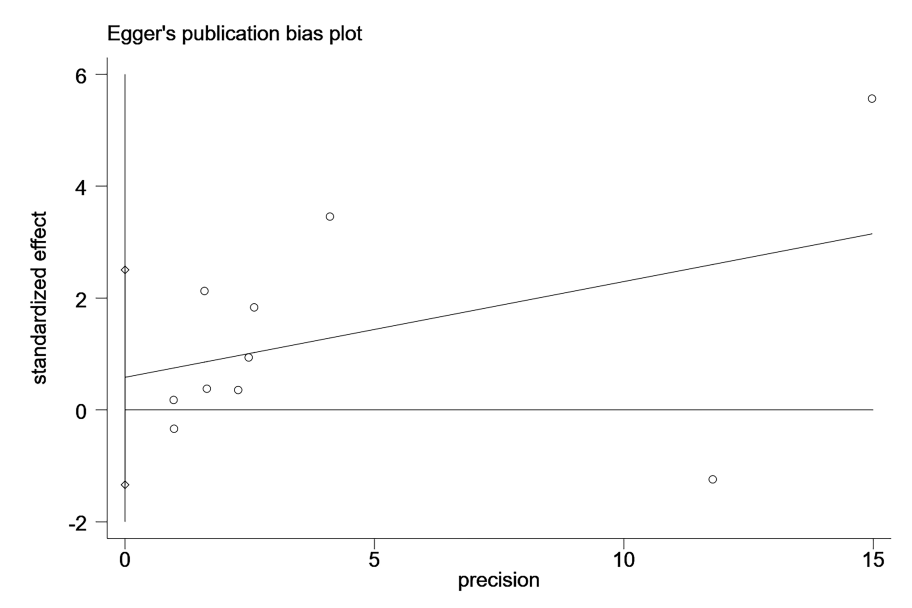


B


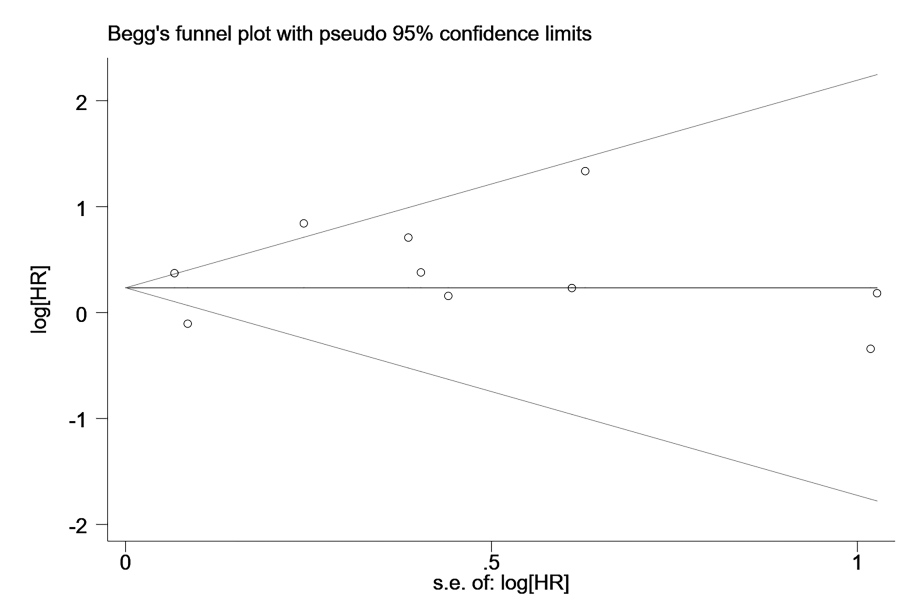


C

**
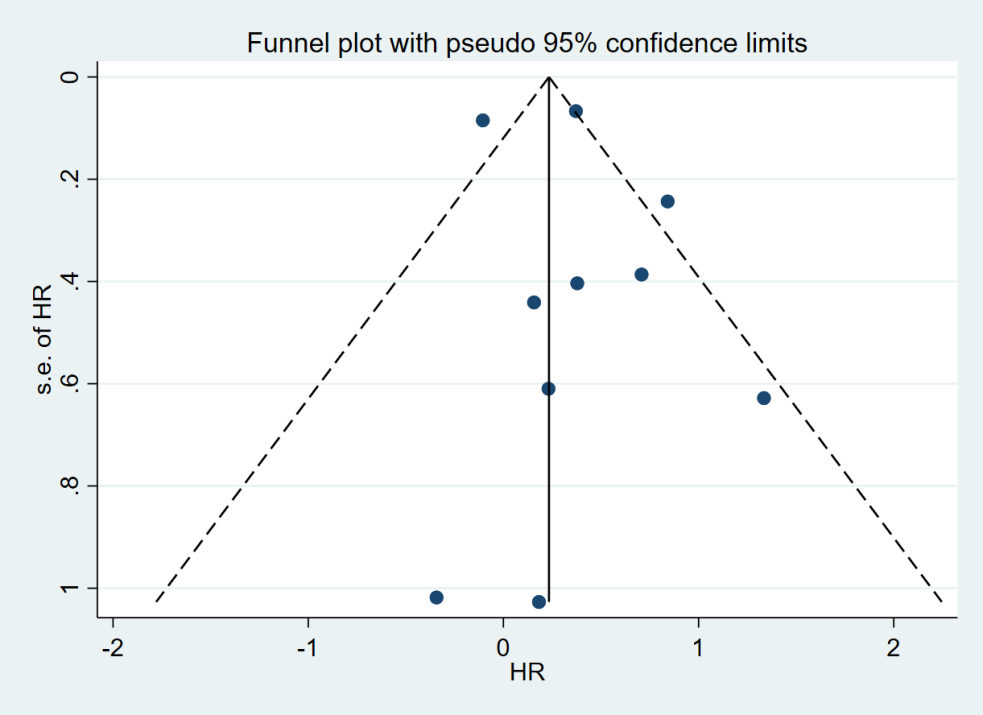
**

**eFigure 4. Forest plot of hazard ratios for the association between isolated diastolic hypertension and all-cause mortality risk**

**
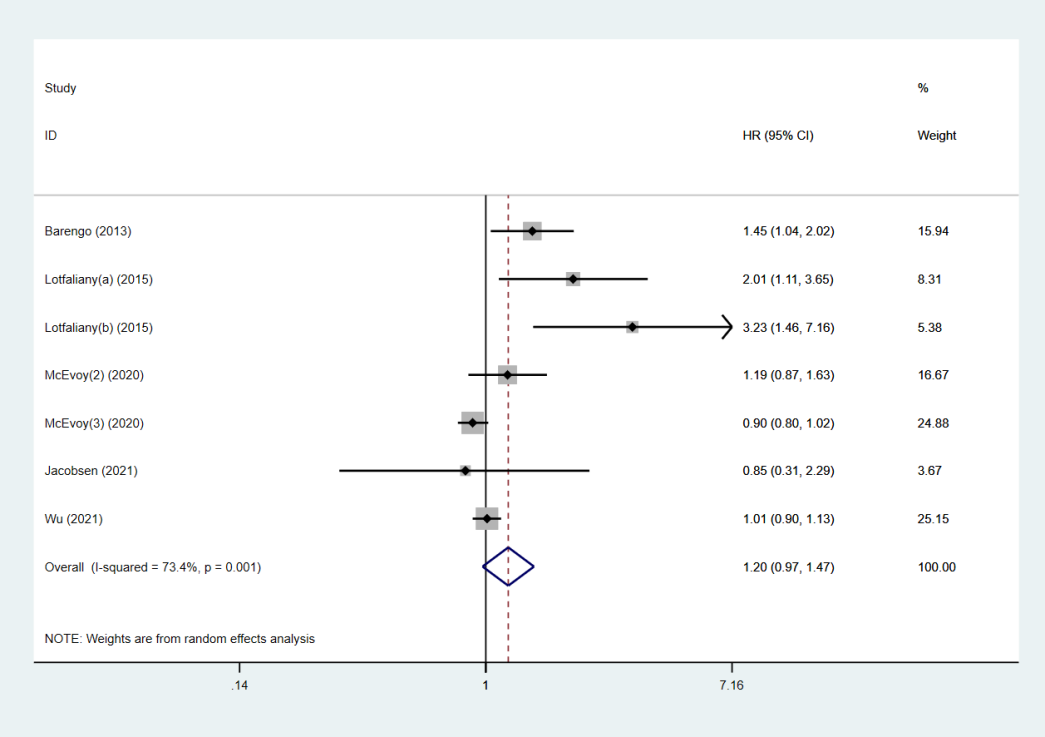
**

**eFigure 5. Egger’s test (A), Begg’s test (B),** **funnel plot (C) and filled funnel plots (D) for studies of isolated diastolic hypertension in relation to the risk of all-cause mortality.**

A


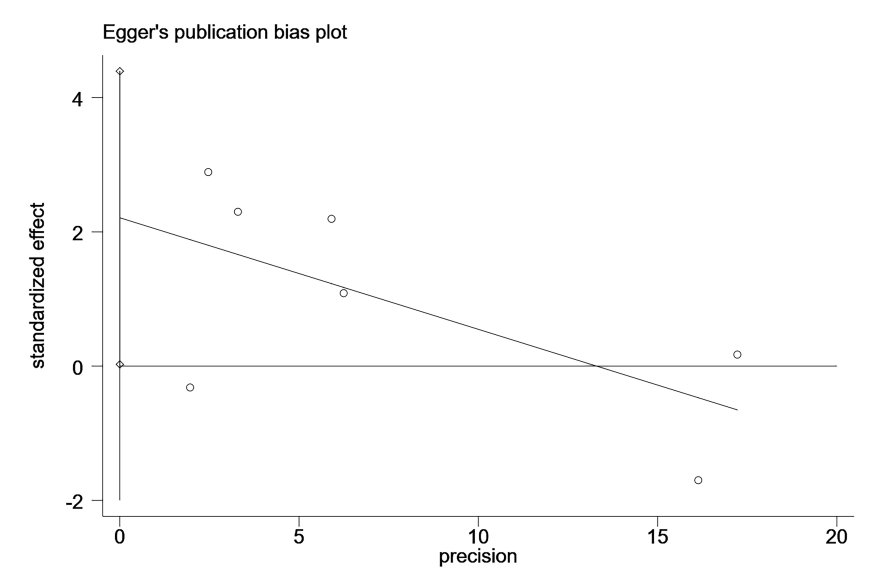


B


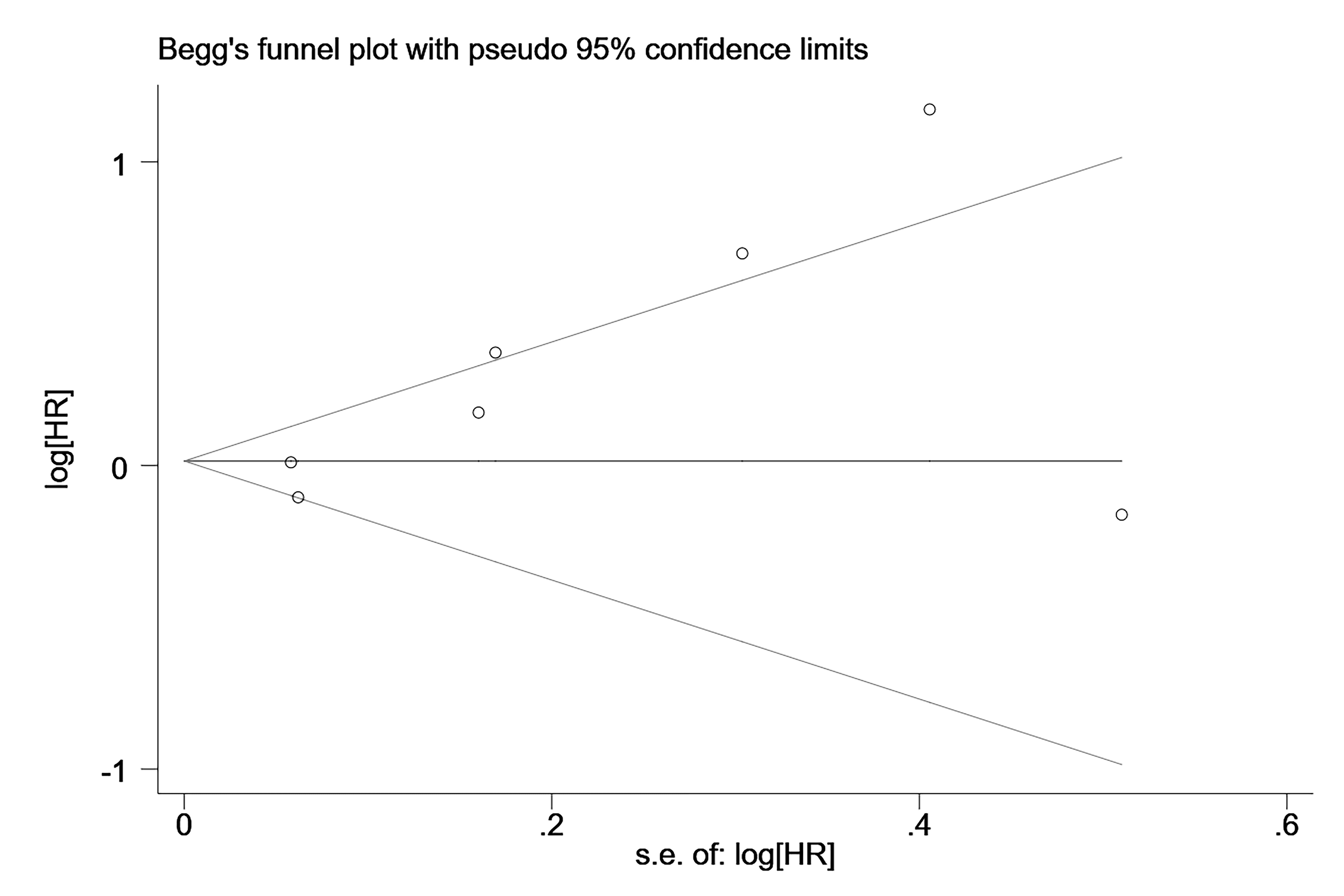


C

**
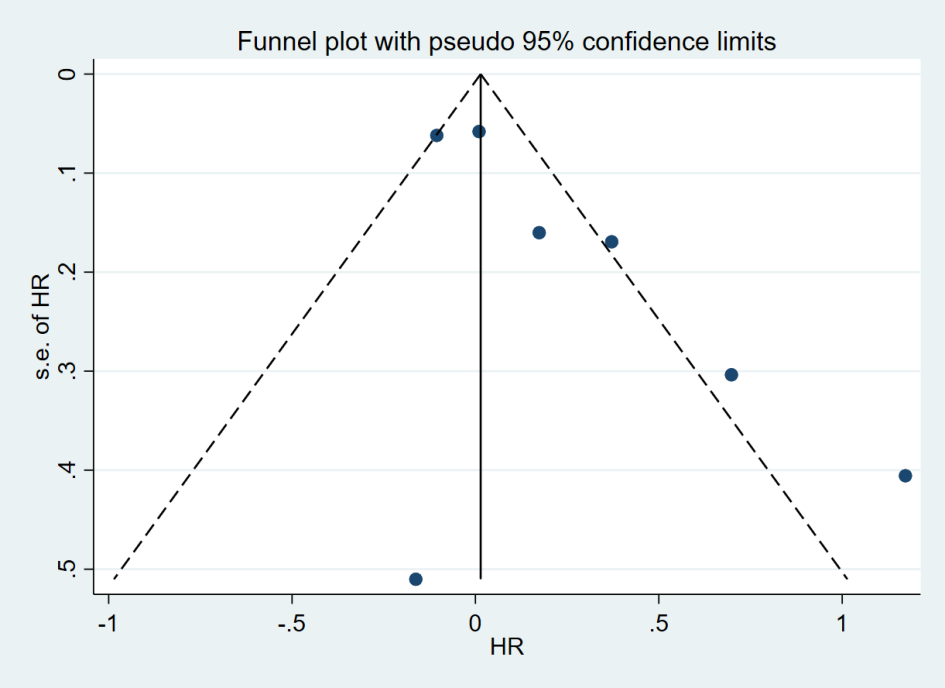
**

D

**
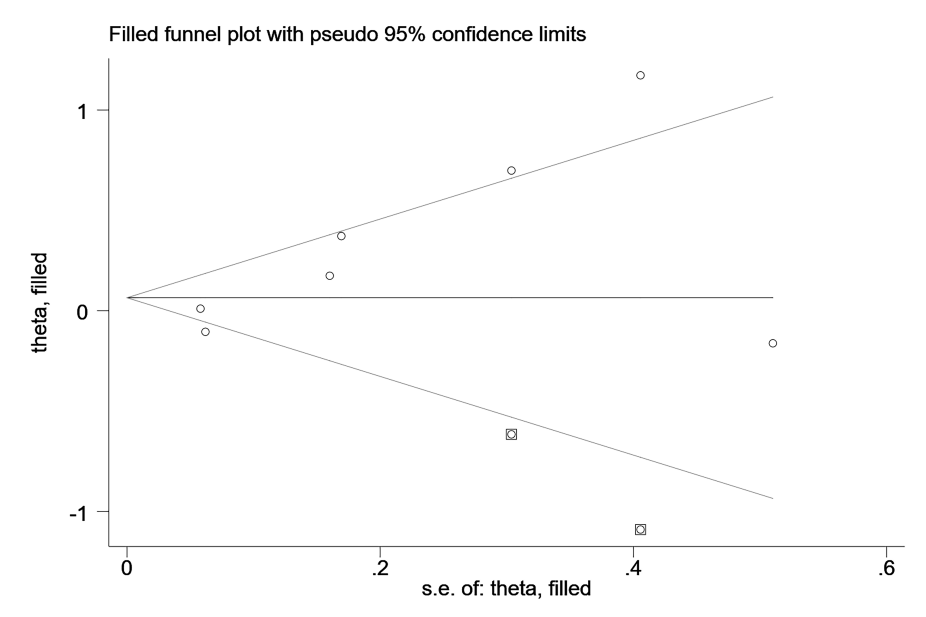
**

**eFigure 6. Forest plot of hazard ratios for the association between isolated diastolic hypertension and all strokes.**


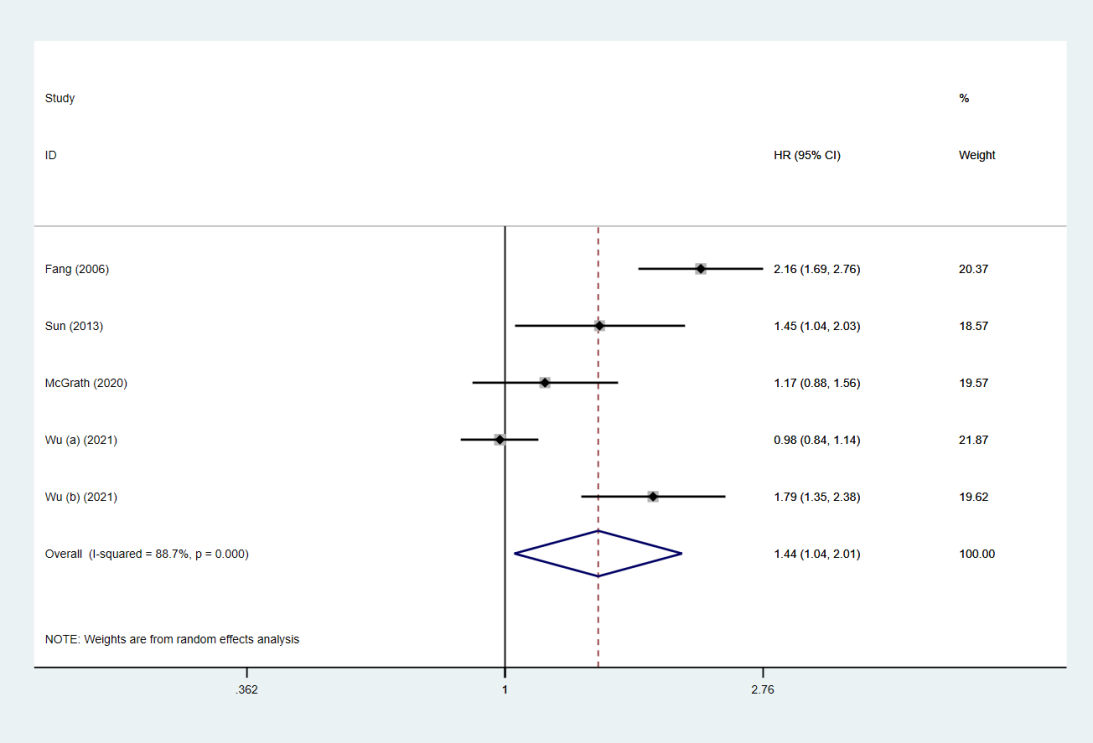


**eFigure 7. Forest plot of hazard ratios for the association between isolated diastolic hypertension and ischemic stroke.**

**
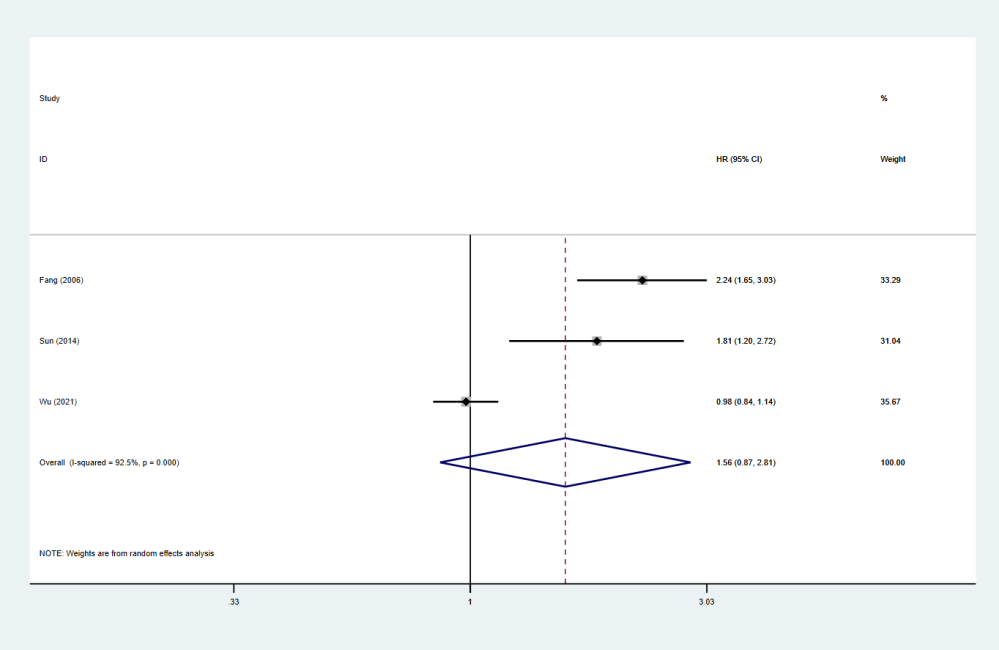
**

**eFigure 8. Forest plot of hazard ratios for the association between isolated diastolic hypertension and hemorrhagic stroke.**


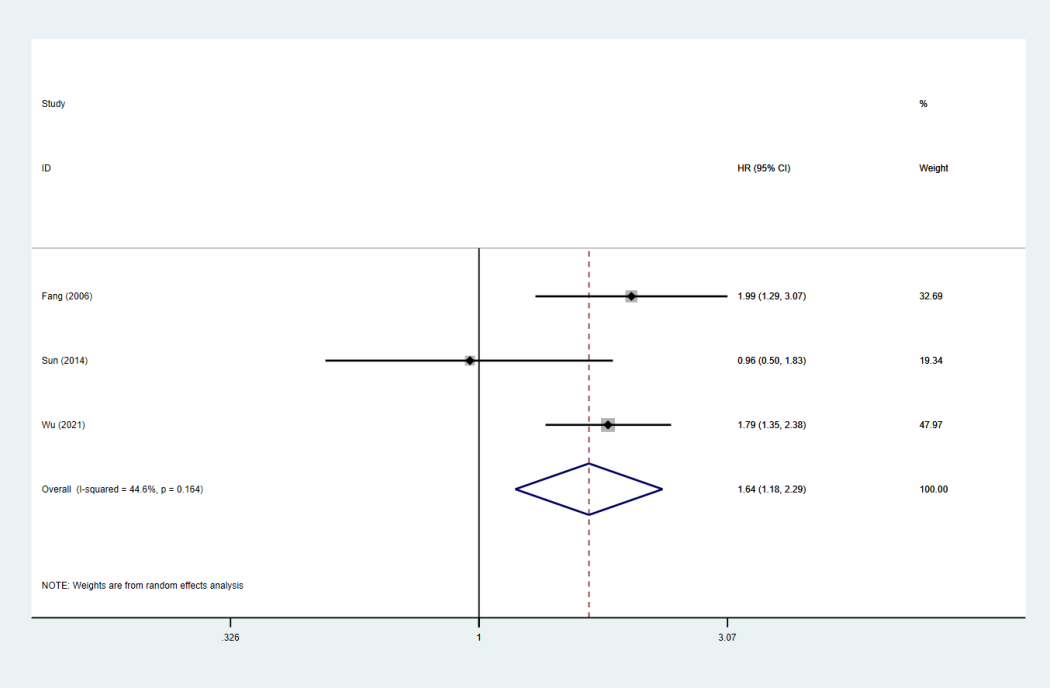


**eTable 1. Newcastle-Ottawa Scale scores and quality assessment of included studies**

|  | Selectoin | | | |  | Outcome | | |  |
| --- | --- | --- | --- | --- | --- | --- | --- | --- | --- |
| Study | Representativeness | Selection | Ascertainment | outcome | Comparability | Assessment | follow-up | Adequacy | Total score |
| Hozawa 2000 | 1 | 1 | 1 | 1 | 1 | 1 | 1 | 1 | 8 |
| Fang 2006 | 1 | 1 | 1 | 1 | 1 | 1 | 1 | 1 | 8 |
| Kelly 2008 | 1 | 1 | 1 | 1 | 0 | 1 | 1 | 1 | 7 |
| Barengo 2013 | 1 | 1 | 1 | 1 | 1 | 1 | 1 | 1 | 8 |
| Carlsson 2013 | 1 | 1 | 1 | 0 | 1 | 1 | 1 | 1 | 7 |
| Niiranen 2014 | 1 | 1 | 1 | 1 | 1 | 1 | 1 | 1 | 8 |
| Sun 2014 | 1 | 1 | 1 | 1 | 1 | 1 | 1 | 1 | 8 |
| Lotfaliany 2015 | 1 | 1 | 1 | 1 | 1 | 1 | 1 | 1 | 8 |
| Hisamatsu 2020 | 1 | 1 | 1 | 1 | 0 | 1 | 1 | 1 | 7 |
| McEvoy(1) 2020 | 1 | 1 | 1 | 1 | 0 | 1 | 1 | 1 | 7 |
| McEvoy(2) 2020 | 1 | 1 | 1 | 1 | 1 | 1 | 1 | 1 | 8 |
| McEvoy(3) 2020 | 1 | 1 | 1 | 1 | 1 | 1 | 1 | 1 | 8 |
| McGrath 2020 | 1 | 1 | 1 | 1 | 0 | 1 | 1 | 1 | 7 |
| Jacobsen 2021 | 1 | 1 | 1 | 1 | 2 | 1 | 1 | 1 | 9 |
| Wu 2021 | 1 | 1 | 1 | 1 | 0 | 1 | 1 | 1 | 7 |

| Study omitted | HR | 95% CI | I^2^ | P |
| --- | --- | --- | --- | --- |
| Kelly 2008 | 1.17 | (1.04, 1.32) | 30.7 | 0.194 |
| Niiranen 2014 | 1.23 | (1.03, 1.46) | 78.9 | ＜0.001 |
| Lotfaliany 2015 | 1.26 | (1.03, 1.54) | 84.2 | ＜0.001 |
| McEvoy(1) 2020 | 1.34 | (1.12, 1.60) | 78.2 | ＜0.001 |
| McGrath 2020 | 1.31 | (1.05, 1.64) | 76.2 | ＜0.001 |
| Jacobsen 2021 | 1.29 | (1.08, 1.54) | 80.9 | ＜0.001 |
| Wu 2021 | 1.31 | (1.05, 1.64) | 77.9 | ＜0.001 |

**eTable 2. Influence analysis excluding one study at a time for the meta-analysis of cohort studies on isolated diastolic hypertension and composite cardiovascular events.**

Abbreviations: CI, confidence interval; HR, hazard ratio.
